# The Role of a Glucosinolate-Derived Nitrile in Plant Immune Responses

**DOI:** 10.3389/fpls.2020.00257

**Published:** 2020-03-10

**Authors:** Hieng-Ming Ting, Boon Huat Cheah, Yu-Cheng Chen, Pei-Min Yeh, Chiu-Ping Cheng, Freddy Kuok San Yeo, Ane Kjersti Vie, Jens Rohloff, Per Winge, Atle M. Bones, Ralph Kissen

**Affiliations:** ^1^Institute of Plant Biology and Department of Life Science, National Taiwan University, Taipei, Taiwan; ^2^Department of Agronomy, National Taiwan University, Taipei, Taiwan; ^3^Faculty of Resource Science and Technology, Universiti Malaysia Sarawak, Kota Samarahan, Malaysia; ^4^Cell, Molecular Biology and Genomics Group, Department of Biology, Norwegian University of Science and Technology, Trondheim, Norway

**Keywords:** secondary metabolites, glucosinolates, nitriles, metabolomics, transcriptomics, plant innate immunity

## Abstract

Glucosinolates are defense-related secondary metabolites found in Brassicaceae. When Brassicaceae come under attack, glucosinolates are hydrolyzed into different forms of glucosinolate hydrolysis products (GHPs). Among the GHPs, isothiocyanates are the most comprehensively characterized defensive compounds, whereas the functional study of nitriles, another group of GHP, is still limited. Therefore, this study investigates whether 3-butenenitrile (3BN), a nitrile, can trigger the signaling pathways involved in the regulation of defense responses in *Arabidopsis thaliana* against biotic stresses. Briefly, the methodology is divided into three stages, (i) evaluate the physiological and biochemical effects of exogenous 3BN treatment on Arabidopsis, (ii) determine the metabolites involved in 3BN-mediated defense responses in Arabidopsis, and (iii) assess whether a 3BN treatment can enhance the disease tolerance of Arabidopsis against necrotrophic pathogens. As a result, a 2.5 mM 3BN treatment caused lesion formation in Arabidopsis Columbia (Col-0) plants, a process found to be modulated by nitric oxide (NO). Metabolite profiling revealed an increased production of soluble sugars, Krebs cycle associated carboxylic acids and amino acids in Arabidopsis upon a 2.5 mM 3BN treatment, presumably via NO action. Primary metabolites such as sugars and amino acids are known to be crucial components in modulating plant defense responses. Furthermore, exposure to 2.0 mM 3BN treatment began to increase the production of salicylic acid (SA) and jasmonic acid (JA) phytohormones in Arabidopsis Col-0 plants in the absence of lesion formation. The production of SA and JA in nitrate reductase loss-of function mutant (*nia1nia2*) plants was also induced by the 3BN treatments, with a greater induction for JA. The SA concentration in *nia1nia2* plants was lower than in Col-0 plants, confirming the previously reported role of NO in controlling SA production in Arabidopsis. A 2.0 mM 3BN treatment prior to pathogen assays effectively alleviated the leaf lesion symptom of Arabidopsis Col-0 plants caused by *Pectobacterium carotovorum* ssp. *carotovorum* and *Botrytis cinerea* and reduced the pathogen growth on leaves. The findings of this study demonstrate that 3BN can elicit defense response pathways in Arabidopsis, which potentially involves a coordinated crosstalk between NO and phytohormone signaling.

## Introduction

Plants are sessile organisms constantly exposed to a wide range of natural enemies, ranging from microbes, small insect herbivores to large herbivores. Once their physical defenses are breached, plants initiate a two-layered innate immune system that allow them to recognize, relay warning signals and mount defense against the invaders (Chisholm et al., 2006; Jones and Dangl, 2006).

The first layer of the innate immune system is primarily regulated by the membrane-bound pattern recognition receptors (PRRs), which enable plants to perceive the pathogen-/microbe- associated molecular patterns (PAMPs/MAMPs) and damage-associated molecular patterns (DAMPs) during a pathogen attack. DAMPs are self-elicitors as they are signal molecules released from damaged or dying plant cells (Roh and Sohn, 2018). The molecular pattern-bound PRRs trigger an innate immune response [PAMP-triggered immunity, (PTI); MAMP-triggered immunity, (MTI); DAMP-triggered immunity; (DTI)] to stop the invasion of pathogens (Heil and Land, 2014; Zipfel, 2014). The pathogens can overcome PTI/MTI/DTI (generally referred as PTI here onward) by deploying specific effector proteins, but may trigger the second layer of the innate immune system.

In the second layer of the innate immune system, plants can recognize the pathogen effector proteins through resistance proteins to activate effector-triggered immunity (ETI) (Rajamuthiah and Mylonakis, 2014). The ETI response is quicker and more intense than PTI. Both PTI and ETI initiate a battery of defense maneuvers, including increase of intracellular calcium and oxidative burst, activation of mitogen-activated protein kinase (MAPK) cascades, activation of transcription factors (such as WRKY family) and defense-related genes regulation (Couto and Zipfel, 2016; Kushalappa et al., 2016). Moreover, ETI response also triggers a hypersensitive response cell death to prevent the spread of pathogens (Vlot et al., 2017). Subsequently, hypersensitive response triggers the synthesis of secondary signal molecules, such as salicylic acid (SA), jasmonic acid (JA), nitric oxide (NO), reactive oxygen species (ROS), and lipid-derived molecules, to induce systemic acquired resistance (Fu and Dong, 2013; Gao et al., 2015).

Phytohormones are small endogenous, low-molecular-weight organic compounds that regulate a plethora of developmental and physiological processes (Bedini et al., 2018). Apart from developmental regulation, the phytohormone signaling cascades of SA, JA, and ethylene have also been associated with the aforementioned two layers of plant immunity, PTI and ETI (Mine et al., 2018). Each of the phytohormones has specific roles yet they can interact/crosstalk with one another either synergistically or antagonistically to execute the most efficient defense responses (Pieterse et al., 2012). Primary metabolites are the basic building blocks of nutrients that are required for normal growth and development of plants. On the flip side, the carbon skeletons and the stored energy of primary metabolites can also be utilized for defense purposes. An example of the alteration of primary metabolism in plants under a pathogen attack involves the post-translational derepression of invertase activity that led to an increased hydrolysis of sucrose into monosaccharides (Bonfig et al., 2010). Meanwhile, amino acids are the precursors of defensive proteins and defense-related secondary metabolites (Coruzzi and Last, 2000).

Secondary metabolites are also involved in plant defense. For example, glucosinolates, which are sulfur and nitrogen-containing plant secondary metabolites found in Brassicaceae. In *Arabidopsis thaliana*, glucosinolates are found in the sulfur-rich cells (Wittstock and Halkier, 2002). Myrosinases (thioglucosidase; EC3.2.1.147), which are present in myrosin cells in neighboring to sulfur-rich cells, will hydrolyze glucosinolates upon pathogen attack or upon mechanical damage that causes the disruption of plant tissue (Bones and Rossiter, 1996). Several glucosinolate hydrolysis products (GHPs), such as isothiocyanate, nitrile, epithionitrile and thiocyanate, can be generated depending on the side chain of their precursor glucosinolates and the presence of specifier proteins (Wittstock and Burow, 2010). Among the known GHPs, isothiocyanates are most studied. Isothiocyanates have been reported to be highly toxic to a broad range of natural enemies of plants including insects, fungi, bacteria and weeds (Halkier and Gershenzon, 2006; Gimsing and Kirkegaard, 2009; Stotz et al., 2011). Isothiocyanates are two edges swords. Not only they are toxic to susceptible pests and diseases, they are also found to be toxic to Arabidopsis at high dosage (Hara et al., 2010). Isothiocyanates also cause other negative effects on plants, such as triggering stomatal closure (Khokon et al., 2011), inducing cell death (Andersson et al., 2015), disrupting microtubules (Øverby et al., 2015a), depleting glutathione (Øverby et al., 2015b), and inhibiting root growth (Urbancsok et al., 2017).

The biological roles of other GHPs, such as nitriles, are not well understood. Nitriles are synthesized from glucosinolates by nitrile-specifier proteins or epithiospecifier proteins, in the presence of myrosinase (Lambrix et al., 2001; Kissen and Bones, 2009). Nitriles are less toxic compared to their corresponding isothiocyanates (Wittstock et al., 2003). It has been demonstrated that an epithiospecifier proteins overexpressing transgenic Arabidopsis, which can produce more nitriles, showed increased resistance to the bacterial pathogen *Pseudomonas syringae pv. tomato* DC3000 and the fungal pathogen *Alternaria brassicicola* (Miao and Zentgraf, 2007). This research indicates that the biological role of glucosinolate-derived nitriles in plants may be involved in disease resistance. Hossain and colleagues previously showed that 3-butenenitrile (3BN) could induce stomatal closure, ROS accumulation and NO production in guard cells of *A. thaliana* (Hossain et al., 2013), which are characteristic processes of DTI. However, to date, there is no study that elucidates the function of 3BN in enhancing the disease tolerance of Brassicaceae plants against pathogens. Therefore, in the present study we chose to investigate the effects of 3BN which is the nitrile-counterpart of the much studied sinigrin-derived allyl-isothiocyanate (Hara et al., 2010; Urbancsok et al., 2017). Among the different Brassicaceae species, *A. thaliana* was used in this study because it is the dicotyledonous model plant. The availability of a wide range of mutant lines and its complete genomic sequence provide extraordinary resources for functional biology studies (Reichelt et al., 2002).

To test the proposed function of 3BN, there were three objectives set out to be achieved in this study, i.e., (i) to evaluate the physiological and biochemical effects of 3BN treatment on Arabidopsis, (ii) to investigate the metabolites triggered by 3BN treatment in Arabidopsis, and (iii) to assess the enhanced disease tolerance of Arabidopsis conferred by 3BN treatment against necrotrophic pathogens.

## Materials and Methods

### Plant Material and Growth Conditions

*Arabidopsis thaliana* wild-type Columbia (Col-0) and nitrate reductase (*nia1nia2*) mutant line were grown in soil in a growth chamber with 16 h light (100 μmol m^–2^ s^–1^)/8 h dark photoperiod at 22°C. The *nia1nia2* mutant line has been described previously (Wilkinson and Crawford, 1993) and the plants were watered with 2.5 mM ammonium nitrate.

### Exposure to 3-Butenenitrile

**FIGURE 1:**
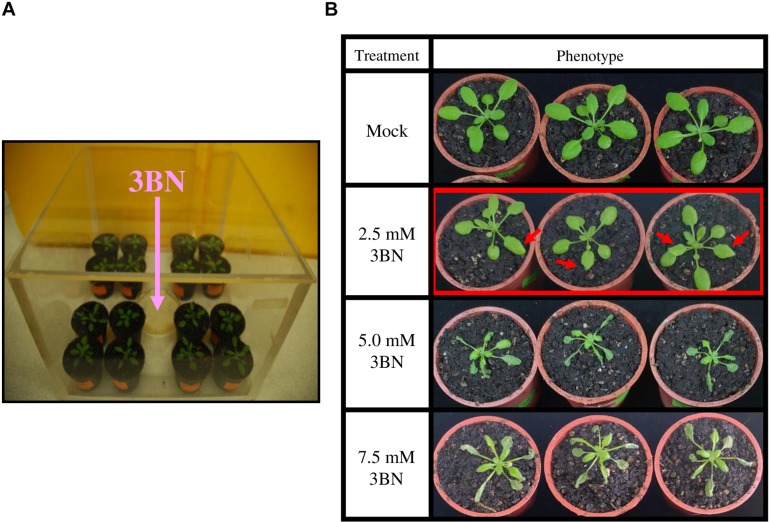
3BN treatment setup and response in Arabidopsis col-0 (wild-type). **(A)** Three weeks old Arabidopsis (wild-type) plants were separately treated with different concentrations of 3BN in closed containers for 24 h. **(B)** The lesion phenotype was observed for 2.5 mM 3BN treatment (red arrows) and severe necrosis was observed for 3BN concentrations ≥ 5.0 mM. Photographs were taken 2 days post-3BN treatment.

3-butenenitrile (3BN, CAS 109-75-1) was purchased from Sigma-Aldrich (122793; 98%). 3BN was freshly diluted in 20 mL of commercial rapeseed oil to different concentrations (2.0, 2.5, 5.0, and 7.5 mM). Three weeks old *A. thaliana* plants were exposed to vapors of the different 3BN concentrations (20 mL in a 9-cm dish, with lid removed) for 24 h in a closed chamber (28.5 cm × 28.5 cm × 19.5 cm) (Figure 1A). The concentration of 3BN vapor emitted from a 2.5 mM 3BN solution into the chamber can be calculated to 1.578 nmol/cm^3^. Plants of different genetic backgrounds were treated together in the same chamber. The setup for control (mock) treatment was identical with plants exposed only to 20 mL rapeseed oil. The effect of the 3BN treatment was compared between the wild-type and *nia1nia2* mutant line. Photographs were taken 2 days post-3BN treatment.

### Analysis of Lesion Formation in 3BN Treated-Plants

Formation of lesion was visualized on rosette leaves 2 days post-3BN vapors treatment by trypan blue staining as described by Koch and Slusarenko (1990) with modifications. Detached leaves were subjected to 2.5 mg/mL of trypan blue and heated in boiling water bath for 1 min. The staining solution was then replaced with chloral hydrate solution (2.5 g/mL) and destained overnight.

Lesion formation was further validated by the electrolyte leakage method using a conductivity meter (Eutech CON 700, Singapore), essentially as described by Dionisio-Sese and Tobita (1998). This experiment was carried out 2 days post-3BN treatment. Electrolyte leakage was calculated using the following formula:

Electrolyteleakage(%)=[Conductivity(unboiledsample)/Conductivity(boiledsample)]×100

### Microarray and Statistical Analysis

The rosette tissue of *A. thaliana* plants (Col-0 and *nia1nia2*) from the mock and 2.5 mM 3BN treatment were harvested after 24 h and immediately flash-frozen in liquid nitrogen and stored at −80°C until further processing. Total RNA was isolated from four biological replicates of each treatment and microarray analysis was performed using the Arabidopsis (V4) Gene Expression Microarray 4 × 44K (Agilent Technology, United States). The details of the procedures were described previously (Kissen et al., 2016).

For the statistical analysis of microarray data, data were preprocessed using the Limma package (version 3.2.3) as implemented in R (Smyth, 2005). Further data normalization and processing were following Kissen et al. (2016). Raw data have been deposited in Gene Expression Omnibus (accession GSE139089).

### Gene Ontology Enrichment and Network Analysis

The identification of differentially expressed genes (DEGs) was set at a cut-off of | log_2_fold change| ≥ 1 and adjusted *p* < 0.01. The identified DEGs from Col-0 and *nia1nia2* were analyzed for Gene Ontology (GO) enrichment analysis using PANTHER classification system^1^ (Mi et al., 2019). Furthermore, the same set of DEGs was selected for network analysis by PATHWAY STUDIO software (Ariadne Genomics, Rockville, MD, United States) and for pathway mapping analysis by MAPMAN (Thimm et al., 2004).

### Metabolite Extraction and Gas Chromatography-Mass Spectrometry (GC-MS) Analysis

Metabolites were extracted from shoot tissue of *A. thaliana* plants (Col-0 and *nia1nia2*) exposed to mock and 2.5 mM 3BN for 24 h (four biological replicates each) and derivatized with MSTFA [N-Methyl-N-(trimethylsilyl)trifluoroacetamide] (Sigma-Aldrich, 69479), followed by GC-MS as described previously (Alipanah et al., 2015). MetAlign software (PRI-Rikilt, Wageningen, The Netherlands) was used for the data integration, normalization, and alignment. Principal component analysis (PCA) was performed using SIMCA software (Umetrics, Sweden). The main goal was to identify metabolites that are produced at different levels (i) between mock and 3BN-treated conditions in each genotype or (ii) between Col-0 and *nia1nia2* plants under the same experimental conditions.

### Phytohormone Analysis

SA, JA, and abscisic acid (ABA) were extracted and analyzed as described by Pan et al. (2010). The liquid chromatography system used for analysis was an ultra-performance liquid chromatography (UPLC) system (ACQUITY UPLC, Waters, Millford, MA, United States). The UPLC system was coupled to a Waters Xevo TQ-S triple quadrupole mass spectrometer (Waters, Milford, MA, United States). Chromatographic separations were performed on an ACQUITY UPLC HSS T3 Column (2.1 × 100 mm, 1.8 μm, Waters, Millford, MA, United States). 13C6- salicylic acid (13C6-SA), d5-jasmonic acid (D5-JA) and d6- abscisic acid (D6-ABA) were used as internal standards. Characteristic mass spectrometry transitions were monitored using negative multiple reaction monitoring (MRM) mode for SA (m/z, 137 > 93), 13C6-SA (m/z, 143 > 99), JA (m/z, 209 > 59), D5-JA (m/z, 214 > 62), ABA (m/z, 263 > 153), and D6-ABA (m/z, 269 > 159). Data acquisition and processing were performed using MassLynx version 4.1 and TargetLynx software (Waters Corp.).

### Plant Pathogen Inoculation and Disease Response Assay

Prior to infection assay, 3BN-treated plants were placed in ventilated fume hood for 30 min to get rid of the 3BN vapor. The infection assay with *Pectobacterium carotovorum* ssp. *carotovorum* (Pcc) was done as described previously (Hsiao et al., 2017) with some modifications. Four rosette leaves from each 3 weeks old plant from the 24 h mock or 2.0 mM 3BN treatment were punctured with a 10 μL tip and inoculated with 10 μL liquid culture of 1 × 10^6^cfu ml^–1^ of Pcc or water (control). The disease symptoms and lesion sizes on leaves were recorded at 16 h post-infection (hpi) and analyzed with Image J. *In planta* bacterial growth assays were performed by counting the bacterial colony-forming unit (CFU) after tissue homogenization and proper dilutions as described by Ishiga et al. (2017).

*Botrytis cinerea* (Bc) infection assay was performed as described previously (Huang et al., 2014) with modifications. Droplets of 10 μL of Bc spore suspension (10^6^ spores ml^–1^) were deposited on each side of the leaf midvein on four rosette leaves from each 3 weeks old plant after the 24 h mock or 2.0 mM 3BN treatment. The infected plants were kept at 100% relative humidity throughout the assay. The disease symptoms and lesion sizes on leaves were recorded at 48 hpi and analyzed with Image J. *In planta* fungal growth assays were performed by analyzing the relative transcript level of *B. cinerea Actin* (*BcActin*) and Arabidopsis *Actin* (*AtActin*) by qRT-PCR. The primers used for qRT-PCR are as described by Nie et al. (2017).

## Results

### Arabidopsis Response to 3BN Treatment

The wild-type plants exhibited a dose-dependent necrosis compared to the mock condition (Figure 1B). Lesions began to appear on rosette leaves upon 2.5 mM 3BN treatment, while more severe necrosis was observed at higher concentrations of 3BN treatments, i.e., ≥ 5.0 mM.

**FIGURE 2:**
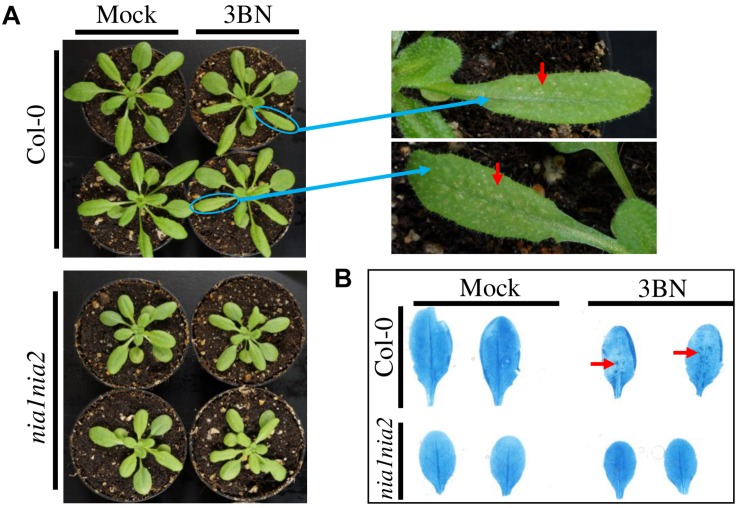
Comparison of physiological and biochemical effects of 2.5 mM 3BN treatment between Arabidopsis Col-0 wild-type and *nia1nia2* mutant. **(A)** Signs of lesion formation (red arrows) were spotted on the wild-type leaves upon 3BN treatment. Photographs were taken 2 days post-3BN treatment. **(B)** Trypan blue staining revealed the presence of dark-blue stained spots, indicating lesion formation in Col-0 wild-type but not *nia1nia2* leaves upon 3BN treatment. Lesion was visualized on rosette leaves 2 days post-3BN vapors treatment.

On the contrary, leaves of the NO-deficient mutant *nia1nia2* did not show any sign of lesions upon the 2.5 mM 3BN treatment (Figure 2A). Consistently, trypan blue staining showed dark-blue staining in the wild-type leaves under the 2.5 mM 3BN treatment but this was not observed in the *nia1nia2* leaves (Figure 2B). The dark-blue staining on the wild-type leaves indicated that 3BN had caused lesion formation.

**FIGURE 3:**
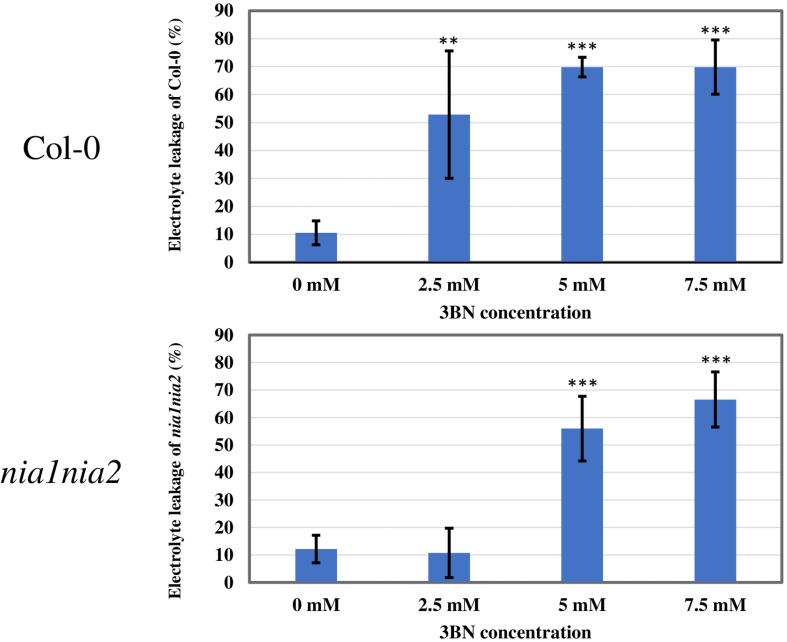
Electrolyte leakage analysis of Arabidopsis Col-0 wild-type and *nia1nia2* mutant when subjected to different concentrations of 3BN treatments. This analysis was carried out 2 days post-3BN treatment. 2.5 mM 3BN treatment caused significant electrolyte leakage in wild-type but not in *nia1nia2* mutant. Values for electrolyte leakage are the average (error bars indicate SD) of six plants. Stars indicate a statistically significant difference (One-way ANOVA, **P < 0.01, ***P < 0.001) to the control treatment.

This finding was further validated by electrolyte leakage analysis (Figure 3). Notably, 2.5 mM 3BN treatment resulted in significant electrolyte leakage in the wild-type plants compared to mock condition but this was not observed in the *nia1nia2* mutant plants. Significant electrolyte leakage only began to happen in the *nia1nia2* mutant plants upon exposure to 5.0 mM 3BN treatment.

### Transcriptomic Responses in Arabidopsis Col-0 and *nia1nia2* Upon 2.5 mM 3BN Treatment

**FIGURE 4:**
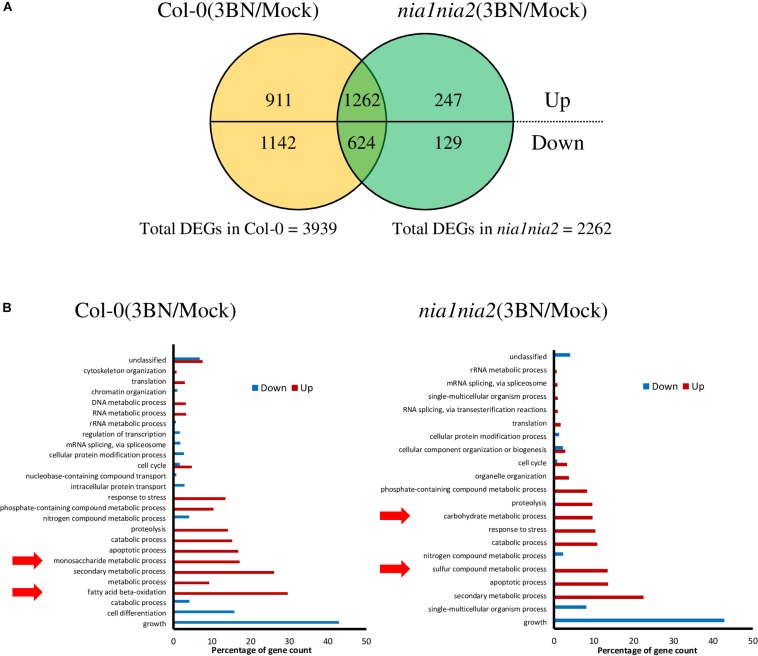
An overview of microarray analysis results for Arabidopsis Col-0 wild-type and *nia1nia2* mutant upon 2.5 mM 3BN treatment for 24 h. **(A)** The comparison of the number of differentially expressed genes (DEGs) under the 3BN treatment between Col-0 and *nia1nia2*. **(B)** GO enrichment analysis of DEGs in Arabidopsis Col-0 wild-type and *nia1nia2* mutant upon 2.5 mM 3BN treatment. Enrichment of monosaccharide metabolic process and fatty acid beta-oxidation was uniquely found in the upregulated genes of the wild-type plants, while enrichment of carbohydrate metabolic process and sulfur compound metabolic process was uniquely found in the upregulated genes of the mutant plants.

A total of 3939 DEGs was identified in Col-0 plants in response to 2.5 mM 3BN treatment of which 2173 genes were upregulated and 1766 genes were downregulated (Figure 4A and Supplementary Table S1). Relatively few DEGs, 2262, were identified in *nia1nia2* plants in response to 2.5 mM 3BN treatment of which 1509 genes were upregulated and 753 genes were downregulated (Figure 4A and Supplementary Table S1). A total of 1262 genes were upregulated in both Col-0 and *nia1nia2* plants, while 624 genes were downregulated in both the wild-type and mutant plants (Figure 4A). In other words, 911 and 247 genes were uniquely upregulated in Col-0 and *nia1nia2* plants, respectively. For downregulated genes, 1142 and 129 genes were unique to the wild-type and mutant plants, respectively (Figure 4A).

GO enrichment analysis of DEGs showed that monosaccharide metabolic process and fatty acid beta-oxidation were enriched by the upregulated genes in the Col-0 plants in response to the 3BN treatment while the enrichment was not detected in the *nia1nia2* plants (Figure 4B). On the other hands, carbohydrate metabolic process and sulfur compound metabolic process were enriched by the upregulated genes in the *nia1nia2* plants in response to the 3BN treatment (Figure 4B).

The network enrichment analysis of Pathway Studio showed that a group of 159 genes was enriched in the plant defense term (*p*-value: 2.08838 E-4) in Col-0 plants (Supplementary Table S2). Among them, a total of 134 (84.3%) defense-related and phytohormone-associated genes were upregulated in the Col-0 plants upon 3BN treatment (Supplementary Table S2). Notably, the expression of SA-biosynthetic genes, *ICS1* (isochorismate synthase 1; At1g74710), *PAD4* (phytoalexin deficient 4; At3g52430), and *EDS1* (enhanced disease susceptibility 1 protein; At3g48090); and that of SA signaling genes, *NPR3*, *NPR4* (non-expressor of pathogenesis-related proteins 3, 4; At5g45110, At4g19660), *TGA3* (TGACG-binding transcription factor 3; At1g22070), *WRKY70* (WRKY DNA-binding protein 70; At3g56400), and *PR5* (pathogenesis-related gene 5; At1g75040), were induced by the 3BN treatment (Supplementary Table S2). In addition, the expression of JA-biosynthetic genes, *LOX1* and *LOX5* (lipoxygenase 1, 5; At1g55020, At3g22400) were also induced by the 3BN treatment (Supplementary Table S2).

### Metabolite Profiling of Arabidopsis Col-0 and *nia1nia2* Upon 2.5 mM 3BN Treatment

**FIGURE 5:**
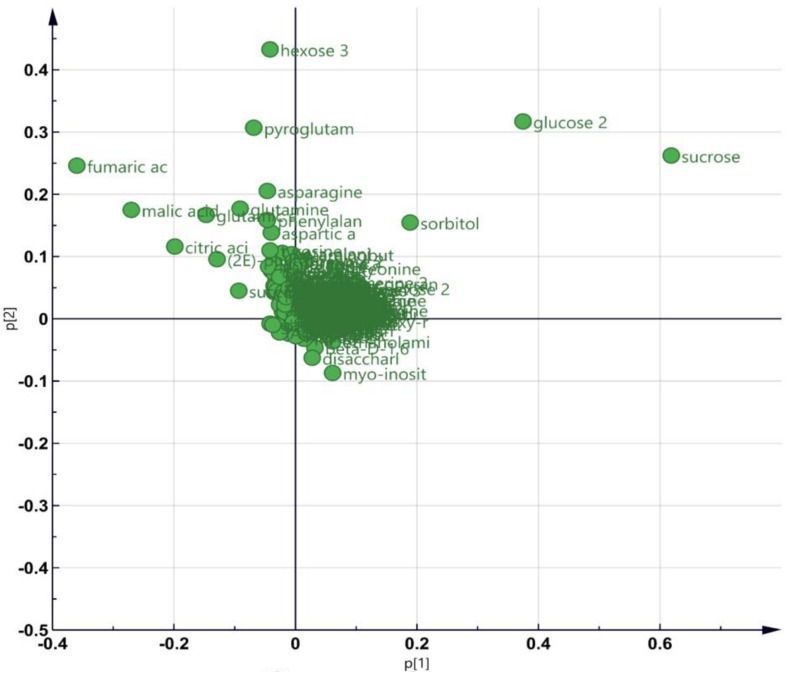
An overview of metabolite profiling data for Arabidopsis Col-0 wild-type and *nia1nia2* mutant upon 2.5 mM 3BN treatment for 24 h. PCA plot depicting the metabolites with variable contents between any combination of paired samples.

As GO enrichment of the DEGs revealed an enrichment of primary metabolic process (Figure 4B), metabolite profiling was carried out (Figure 5). PCA plot of samples shows that the four biological replicates of the four samples (i.e., Col-0_mock, Col-0_3BN, *nia1nia2*_mock, *nia1nia2*_3BN) were clustered according to their own groupings, indicating that the samples were of good quality for metabolite profiling (Supplementary Figure S1 and Supplementary Table S3). The PCA plot of metabolites depicts that the four samples can be differentiated mainly based on the content of various types of sugars, carboxylic acids and amino acids (Figure 5).

**FIGURE 6:**
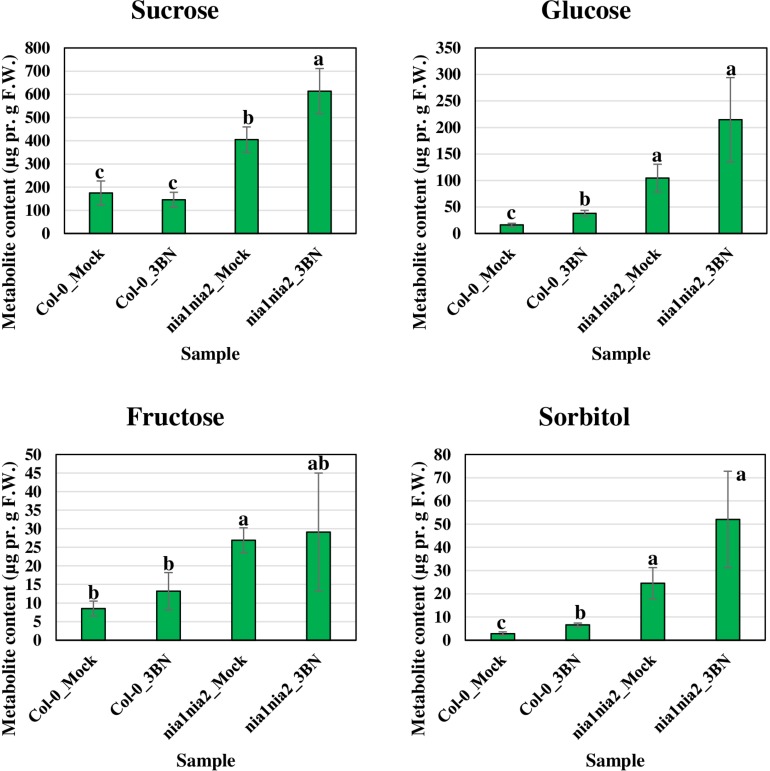
Metabolite profiling of soluble sugars (including sorbitol, a sugar alcohol) for Arabidopsis Col-0 wild-type and *nia1nia2* mutant upon 2.5 mM 3BN treatment for 24 h. For sucrose, One-way ANOVA and Tukey *post hoc* test were used with different letters denoting a significant difference (*P* < 0.05). For glucose, fructose and sorbitol, where the homogeneity of variance assumption could not be fulfilled, Welch one-way test and pairwise *t*-test were used with different letters denoting a significant difference (*P* < 0.05).

**FIGURE 7:**
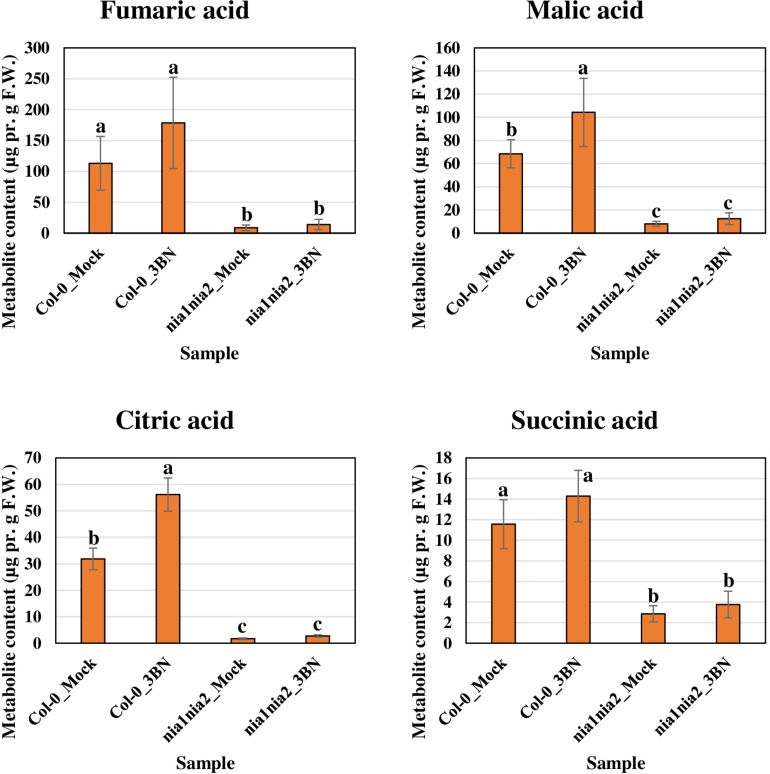
Metabolite profiling of Krebs cycle-associated carboxylic acids for Arabidopsis Col-0 wild-type and *nia1nia2* mutant upon 2.5 mM 3BN treatment for 24 h. One-way ANOVA and Tukey *post hoc* test with different letters denoting a significant difference (*P* < 0.05).

**FIGURE 8:**
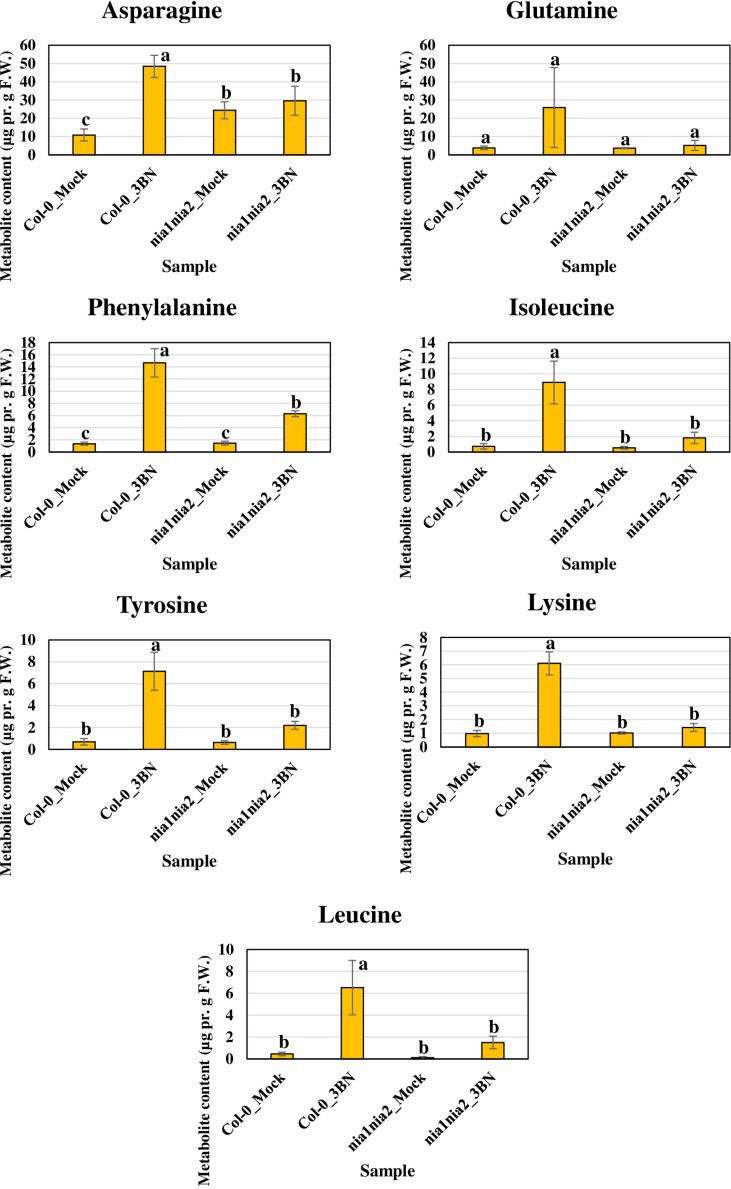
Metabolite profiling of amino acids for Arabidopsis Col-0 wild-type and *nia1nia2* mutant upon 2.5 mM 3BN treatment for 24 h. One-way ANOVA and Tukey *post hoc* test with different letters denoting a significant difference (*P* < 0.05).

The content of the metabolites in all four samples showed three important trends. Firstly, soluble sugars, such as sucrose, glucose and fructose, as well as sorbitol (sugar alcohol) were produced at lower levels in the Col-0 leaves than in the *nia1nia2* leaves under mock condition (Figure 6). The 3BN treatment generally increased the production of these soluble sugars and sorbitol in wild-type and mutant plants. Particularly, sucrose content was increased by the 3BN treatment in the *nia1nia2* leaves, while glucose and sorbitol were increased by the same treatment in the Col-0 leaves (Figure 6). Next, four carboxylic acids that are intermediate compounds in the Krebs cycle, namely fumaric acid, malic acid, citric acid and succinic acid were produced at higher levels in the Col-0 leaves than in the *nia1nia2* leaves under mock condition (Figure 7). The 3BN treatment generally increased the production of these carboxylic acids in Col-0 plants with significant difference for malic acid and citric acid (Figure 7). The third observation was that the 3BN treatment increased the production of six amino acids in Col-0 to a greater extent than in *nia1nia2* plants (Figure 8).

### Changes of Phytohormone Production in Arabidopsis Col-0 and *nia1nia2* Upon 2.0 and 2.5 mM 3BN Treatments

**FIGURE 9:**
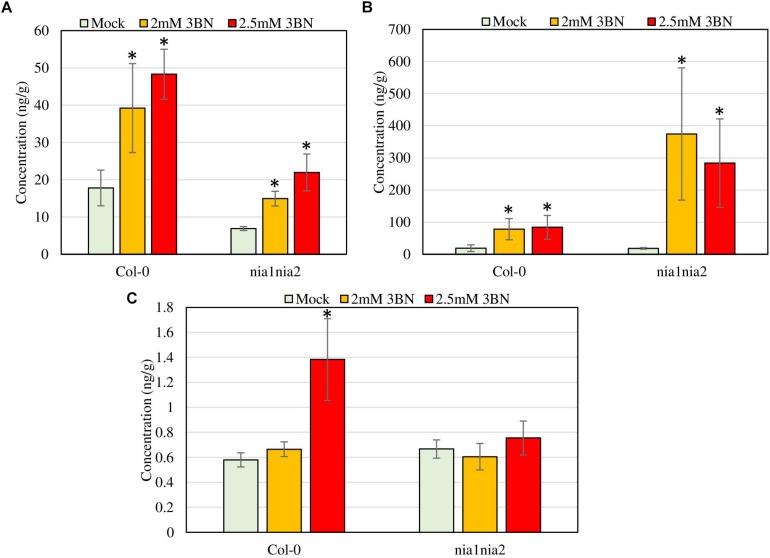
Phytohormone profiling of Arabidopsis Col-0 wild-type and *nia1nia2* mutant upon 2.0 and 2.5 mM 3BN treatments for 24 h. **(A)** SA, **(B)** JA, and **(C)** ABA (*t*-test, *P < 0.05, *n* = 3 per sample).

Based on the microarray data, the expression of genes associated with phytohormone biosynthesis and signaling were upregulated in Arabidopsis Col-0 wild-type in response to 2.5 mM 3BN treatment (Supplementary Table S2). This finding suggests the involvement of phytohormone signaling in the 3BN-induced innate immune responses. Meanwhile, electrolyte leakage analysis was repeated with the inclusion of 2.0 mM 3BN treatment in the second trial (Supplementary Figure S2). Both trials showed consistent results where 2.5 mM 3BN treatment only caused significant electrolyte leakage in the Col-0 plants compared to mock condition but not in the *nia1nia2* mutant plants (Figure 3 and Supplementary Figure S2). Interestingly, 2.0 mM 3BN treatment did not cause electrolyte leakage in both the wild-type and mutant plants (Supplementary Figure S2). Therefore, phytohormone production was measured for Col-0 and *nia1nia2* plants treated with 2.0 and 2.5 mM 3BN treatments in comparison with mock condition (Figure 9).

Ultra-performance liquid chromatography (UPLC) analysis demonstrated that at 2.0 mM 3BN treatment, Arabidopsis Col-0 wild-type had an increase in the production of SA (2.2-fold) and JA (4.1-fold) while 2.5 mM 3BN treatment led to an increased production of SA (2.7-fold), JA (4.4-fold), and ABA (2.4-fold) in comparison with the mock condition (Figure 9). On the other hand, for *nia1nia2* mutant, the 2.0 mM 3BN treatment induced the production of SA (2.2-fold) and JA (20.3-fold) in consonance with the 2.5 mM 3BN treatment that increased the production of SA (3.2-fold) and JA (15.4-fold) (Figure 9). Although 3BN treatments increased the production of SA in both Arabidopsis Col-0 and *nia1nia2* plants, SA concentration in Col-0 was higher than in *nia1nia2* plants under mock, 2.0 and 2.5 mM 3BN treatment conditions (Figure 9A). Nevertheless, significant difference of SA concentration between Col-0 and *nia1nia2* plants was detected only under 2.5 mM 3BN treatment condition (*t*-test, ^∗∗^*P* < 0.01, *n* = 3 per sample). On the other hand, although 3BN treatments increased the production of JA in both Arabidopsis Col-0 and *nia1nia2* plants, JA in *nia1nia2* was induced more by 3BN treatments than in Col-0 (Figure 9B). However, the difference of JA concentration between Col-0_2.0 mM 3BN and *nia1nia2*_2.0 mM 3BN and between Col-0_2.5 mM 3BN and *nia1nia2*_2.5 mM 3BN was not statistically significant, presumably due to large within-group variation.

### Exposure to 2.0 mM 3BN Enhanced Arabidopsis Tolerance to Necrotrophic Pathogens

**FIGURE 10:**
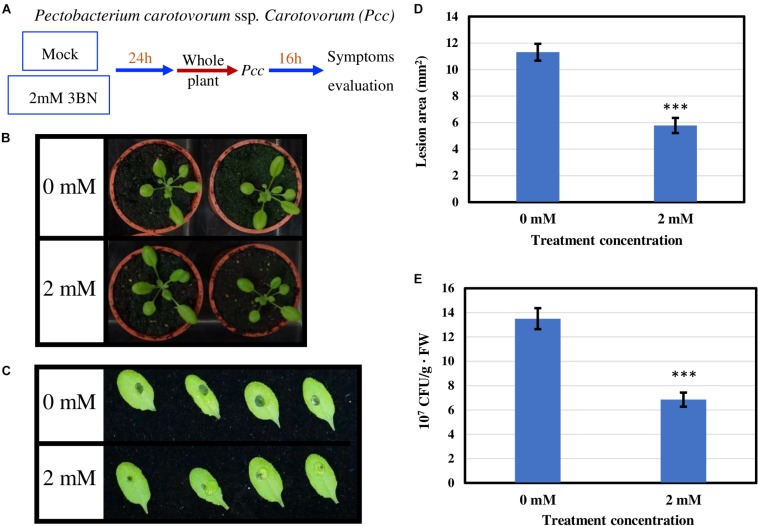
Pathogen assays of Arabidopsis Col-0 with the necrotrophic bacterium *Pectobacterium carotovorum* ssp. *Carotovorum* (Pcc). **(A)** Arabidopsis Col-0 was challenged with the bacterium after having been exposed to a mock or 2.0 mM 3BN treatment for 24 h. **(B)** Representative pictures of plants exposed to a mock or 2.0 mM 3BN treatment for 24 h before pathogen inoculation. **(C)** Representative pictures of rosette leaves showing lesions post 16 h of inoculation with Pcc. **(D)** The lesion area at 16 h post-infection (*t*-test, ***P < 0.001, *n* = 32 leaves per treatment, with four batch data). **(E)** The bacterial population estimated by CFU at 16 h post-infection (*t*-test, ***P < 0.001, *n* = 32 leaves per treatment, with three batch data).

**FIGURE 11:**
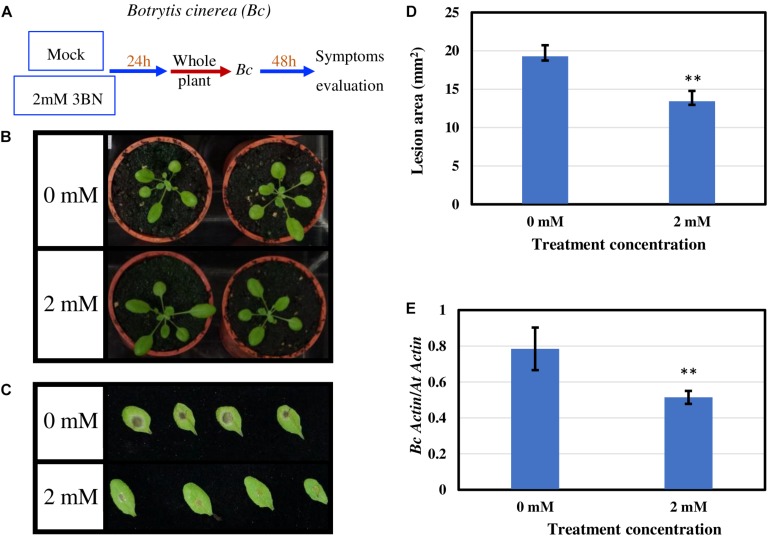
Pathogen assays of Arabidopsis Col-0 with the necrotrophic fungus *Botrytis cinerea* (Bc). **(A)** Arabidopsis Col-0 was challenged with the fungus after having been exposed to a mock or 2.0 mM 3BN treatment for 24 h. **(B)** Representative pictures of plants exposed to a mock or 2.0 mM 3BN treatment for 24 h before pathogen inoculation. **(C)** Representative pictures of rosette leaves showing lesions post 48 h of inoculation with Bc. **(D)** The lesion area at 48 h post-infection (*t*-test, **P < 0.01, *n* = 48 leaves per treatment). **(E)** Quantification of Bc growth at 48 h post-infection estimated by relative transcript levels of *BcActin* and *AtActin* using qRT-PCR (*t*-test, **P < 0.01, *n* = 5 plants per treatment).

To examine whether 3BN-induced innate immune responses can enhance the disease tolerance of Arabidopsis, 2.0 mM 3BN treatment was selected mainly for three reasons, (i) 2.0 mM 3BN treatment did not cause electrolyte leakage on the leaves of Arabidopsis (Supplementary Figure S2), (ii) yellow chlorosis and lesion began to appear on the Col-0 leaves upon 2.5 mM 3BN treatment (Figures 1–3), and (iii) the production of phytohormones such as SA and JA began to be induced in Col-0 leaves upon 2.0 mM 3BN treatment (Figure 9). Three weeks old Arabidopsis Col-0 plants were first exposed to the mock and 2.0 mM 3BN treatments and then subjected to infection assays of *Pectobacterium carotovorum* ssp. *carotovorum* or *Botrytis cinerea*. Interestingly, compared with mock condition, exogenous application of 2.0 mM 3BN was effective in reducing the lesion size and pathogen growth on the leaves of Arabidopsis Col-0 in both of the bacterial (Figure 10) and fungal (Figure 11) infection assays.

## Discussion

### The Involvement of NO in Lesion Formation of Arabidopsis Upon 3BN Recognition

One of the earlier responses of PTI and ETI in plants involves the rapid accumulation of oxygen-derived free radicals such as ROS and NO (Torres et al., 2006). It has been well known that ROS and NO are important signal molecules in plant defense against pathogens, implicated in (i) cross-linking of cell wall proteins, (ii) alteration of membrane permeability and ion fluxes, (iii) hypersensitive response, (iv) systemic acquired resistance, and (v) the production of phytoalexins (Wojtaszek, 1997; Romero-Puertas et al., 2004). The GHPs of interest in this study, 3BN, may play a role in the oxidative burst phenomenon.

In this study, 2.5 mM 3BN treatment caused lesion formation on the leaves of 3 weeks old Arabidopsis Col-0 wild-type while the same treatment did not cause any symptom on the leaves of NO-deficient *nia1nia2* mutant plants (Figures 2, 3). To interpret this result, it is important to understand the various NO production mechanisms in plants. Generally, NO can be produced via enzymatic and non-enzymatic pathways (Gupta et al., 2011). Nitrate reductase and NO synthase-like (NOS-like) activities are the main sources of NO production in plants under normal physiological conditions (Mur et al., 2013). The nitrate reductase-defective double mutant, *nia1nia2*, accumulates a lower endogenous NO level than the Col-0 wild-type (Zhao et al., 2009, 2016). Therefore, it can be deduced that the relatively low endogenous NO level in *nia1nia2* plants was not sufficient to cause lesion formation upon exposure to the 2.5 mM 3BN treatment. This result demonstrates that NO plays a fundamental role in triggering lesion formation upon 3BN recognition.

NO was initially reported in mammals as a key redox-active signal molecule responsible for inflammatory and innate immune responses (Schmidt and Walter, 1994; Stamler, 1994). Since then, a growing body of evidence showed that NO is also playing a pivotal role in plant defense signaling as the NOS-like activity (NOS is a NO biosynthetic enzyme in metazoans) and the secondary messenger molecules in NO-mediated signaling pathways of mammalian cells, such as cyclic guanosine monophosphate (cGMP) and cyclic adenine dinucleotide phosphate ribose (cADPR), are also present in plants (Klessig et al., 2000; Neill et al., 2003; Astier et al., 2018). During pathogen attack, NO is known to regulate hypersensitive response in plants via S-nitrosylation of thiol-containing proteins (post-translational protein modification). For example, NO-mediated S-nitrosylation inhibited the activity of aconitase enzyme in the Krebs cycle (Gardner et al., 1997). The inhibition action resulted in an increased level of cellular iron which together in the presence of NO might contribute to cell death (Navarre et al., 2000). The targets of NO-mediated S-nitrosylation also include antioxidative enzymes such as peroxiredoxin II E (Romero-Puertas et al., 2007), catalase (Lin et al., 2012), and ascorbate peroxidase (de Pinto et al., 2013). Inhibition of these enzymes leads to increased ROS levels presumably responsible for plant cell death. In agreement, the expression of genes encoding two NADPH oxidases, also known as respiratory burst oxidase homologs (RBOHs), i.e., RBOH C (At5g51060), and RBOH F (At1g64060), which produce ROS, was induced by the 2.5 mM 3BN treatment in both Col-0 and *nia1nia2* plants (Supplementary Table S1). Conversely, the accumulation of NO during pathogen attacks was reported to be involved in a negative feedback loop by negatively regulating ROS production, via S-nitrosylation of NADPH oxidase, in order to prevent excessive cell death (Yun et al., 2011).

On the other hand, the electrolyte leakage analysis revealed lesion formation in the *nia1nia2* mutant at a 3BN treatment of ≥5.0 mM (Figure 3). As the NO-deficient phenotype of *nia1nia2* plants is accounted for by the mutation of nitrate reductase 1 and 2 (Zhao et al., 2016), the observed lesion formation of *nia1nia2* when exposed to higher 3BN concentrations could be due to ROS accumulation and/or the eventual build-up of NO catalyzed by other NO biosynthetic enzymes such as NOS-like activity in the mutant plants. It has been reported that NO can cooperate with H_2_O_2_ in triggering hypersensitive response cell death during incompatible plant-pathogen interactions (Yoshioka et al., 2011). Whether NO allies with ROS to activate the programmed cell death of Arabidopsis upon 3BN treatment still remains to be elucidated.

### The Production of Soluble Sugars, Krebs Cycle-Associated Carboxylic Acids, and Amino Acids Is Induced in Arabidopsis by 2.5 mM 3BN Treatment

To optimize fitness under the changing environmental conditions, plants utilize a plethora of signal molecules and the associated-signaling pathways to plastically adjust their metabolism so that priority is given for development under favorable condition while defense responses are activated under unfavorable conditions (Malinovsky et al., 2017). Simple sugars and amino acids are primary metabolites that are indispensable for normal growth, development and reproduction of plants. By the same token, both of these primary metabolites have also been linked to plant stress responses. For instance, sugars were reported to interact with ROS signaling pathways where elevated levels of sugars can either stimulate or repress ROS production (Couée et al., 2006). The amino acid phenylalanine is the first precursor in phenylpropanoid pathway which gives rise to the formation of many important defense-related secondary metabolites (Fraser and Chapple, 2011).

Our metabolite profiling showed that the production of monosaccharide (glucose) and its derivative (sorbitol) in Arabidopsis Col-0 plants was increased by the 2.5 mM 3BN treatment, while the production of disaccharide (sucrose) in *nia1nia2* plants was increased by the same treatment (Figure 6). This observation indicates that sugar metabolism is differentially regulated between the Col-0 and *nia1nia2* plants under the 3BN treatment. By mapping the DEGs from Col-0 and *nia1nia2* plants to the metabolic pathways in MAPMAN, we noticed that a higher number of genes associated with (i) starch and sucrose metabolism and (ii) glycolysis was up-regulated by 3BN treatment in the Col-0 in comparison with the *nia1nia2* plants (Supplementary Figure S3 and Supplementary Table S4). Based on this finding, it can be inferred that sucrose degradation and glycolysis were concomitantly activated by the 2.5 mM 3BN treatment in the Col-0 plants. This could justify the metabolite results where 2.5 mM 3BN treatment did not increase the sucrose level but increased the levels of glucose and sorbitol in the Col-0 plants (Figure 6). Conversely, as sucrose degradation and glycolysis were not activated in the *nia1nia2* plants by the same 3BN treatment, an increase in the sucrose level was observed instead for the levels of monosaccharides and sorbitol (Figure 6). A previous study of comparative metabolomic analysis reported an increased endogenous content of soluble sugars upon melatonin treatment and *Pseudomonas syringae pv. tomato* (*Pst*) DC3000 infection in Arabidopsis Col-0 (Qian et al., 2015). The study also demonstrated that exogenous pre-treatment of soluble sugars could reduce the bacterial spread in Arabidopsis Col-0 but not in SA- and NO-deficient mutants (Qian et al., 2015).

The production of Krebs cycle-associated carboxylic acids, namely malic and citric acids was induced by the 3BN treatment only in the Arabidopsis Col-0 plants (Figure 7). Interestingly, apart from the observed induction of sucrose degradation and glycolysis, genes related to the carboxylic acid-producing processes in the Krebs cycle were also up-regulated by the 2.5 mM 3BN treatment in the Col-0 plants (Supplementary Figure S3 and Supplementary Table S4). Meanwhile, no induction of Krebs cycle-associated genes was observed in the *nia1nia2* plants treated with 2.5 mM 3BN (Supplementary Figure S3). As mounting defense response is an energy-consuming process, it is not surprising that the Krebs cycle, the cellular energy production pipeline, is involved. Furthermore, the cycle also functions in producing the precursors of certain amino acids. In line with this notion, six amino acids, including asparagine, phenylalanine, isoleucine, tyrosine, lysine and leucine exhibited increased production in Arabidopsis Col-0 under 3BN treatment (Figure 8). This increased supply of amino acids could be channeled for the production of defensive proteins or defense-related secondary metabolites (Coruzzi and Last, 2000). It is noteworthy that the increased production of amino acids seen in Arabidopsis Col-0 plants upon 3BN treatment was compromised incompletely in *nia1nia2* plants (Figure 8). This finding can be partly attributed to the two mutated nitrate reductases in *nia1nia2* plants that negatively affect the nitrate reduction/assimilation, which is critical for the production of amino acids (Zhao et al., 2016).

Interestingly, a recent study reported that an acute dose of exogenous NO gas caused momentary metabolic changes in Arabidopsis at 6 h after treatment. The metabolic changes included elevated content of sugars, Krebs cycle carboxylic acids and amino acids along with accumulation of polyamines, fatty acids, phospholipids and nucleic acids (León et al., 2016). The transient effect with a metabolic change at 6 h, but not 24 h as reported in our study, might be due to the fact that NO was treated directly in the study while it may take some time for 3BN to act and generate NO in our study. Nevertheless, based on León et al. (2016), the 3BN-induced NO production is very likely to result in the observed concomitant activation of sucrose degradation, glycolysis and Krebs cycle in the Col-0 plants (Supplementary Figure S3). Besides that, the reported effects of NO on metabolic changes can also be depicted in our metabolite profiling results under mock condition. For example, the higher endogenous NO level in Col-0 plants compared with *nia1nia2* plants could account for the lower levels of soluble sugars but higher carboxylic acid levels in Col-0 plants compared with *nia1nia2* plants under the mock condition (Figures 6, 7). This finding suggests that, to a certain degree, the concomitant activation of sucrose degradation, glycolysis and Krebs cycle has happened in the Col-0 plants under the mock condition. Given the co-induction of these processes under the mock condition, the amino acid levels in the Col-0 plants were still comparable with those measured in the *nia1nia2* plants (Figure 8). A possible explanation is that the efficiency of conserving metabolic resources, such as carbon, nitrogen and energy, is lower if Arabidopsis Col-0 plants accumulate higher amount of amino acids under the mock condition. Conserving metabolic resources for amino acid synthesis is critical because amino acids are the building blocks for various important molecules, including proteins, vitamins, nucleotides and secondary metabolites (Arnold et al., 2015). Thus, increased amino acid biosynthesis, presumably mediated by NO, was only detected when Col-0 plants were treated with 2.5 mM 3BN treatment (Figure 8).

### The Production of SA and JA Is Induced in Arabidopsis by 2.0 mM 3BN Treatment in the Absence of Lesion Formation

Phytohormones are small endogenous, low-molecular-weight regulators of diverse growth, development and physiological processes (Checker et al., 2018). Besides that, they have also been attributed to the modulation of biotic and abiotic stress responses whereby crosstalks exist between the two networks (Ku et al., 2018). Under a stressful condition, antagonistic or synergistic crosstalks also happen between different phytohormones in plants (Ohri et al., 2015). Though complex, the crosstalks between phytohormone signaling cascades are deemed fundamental in fine-tuning the allocation of resources to the most appropriate defense mechanisms (Pieterse et al., 2012).

JA signaling is well known in triggering defense responses against necrotrophic pathogens (Mei et al., 2006), while SA signaling is effective against biotrophs/hemibiotrophs (Sticher et al., 1997). The finding that 2.0 mM 3BN treatment could increase the production of SA and JA is promising because it might enhance the broad-spectrum disease tolerance of Arabidopsis. As the fold-change was higher for JA than SA it remains to be elucidated whether the 3BN-induced defense responses are more effective against the necrotrophic pathogens. In addition, the 2.0 mM 3BN concentration did not cause lesion formation in the Col-0 plants as opposed to 2.5 mM 3BN treatment (Figures 10B, 11B and Supplementary Figure S2). As NO is responsible for the 2.5 mM 3BN-induced lesion formation in the Col-0 plants (as discussed in section The Involvement of NO in Lesion Formation of Arabidopsis Upon 3BN Recognition), it is very likely that the endogenous NO level accumulated in the Col-0 leaves under the 2.0 mM 3BN treatment condition is not sufficient for lesion formation. Therefore, we can infer that the 2.0 mM 3BN treatment establishes a primed state in Arabidopsis Col-0 plants by inducing the production of the phytohormones SA and JA in the absence of lesion formation.

The 2.5 mM 3BN treatment led to NO-mediated lesion formation and induced ABA levels only in Arabidopsis Col-0 plants, while *nia1nia2* plants have compromized NO levels and ABA induction. This observation could indicate a positive regulation between NO and ABA production in leaves. The physiological responses promoted by ABA have been well characterized in which ABA is known to induce callose accumulation and stomatal closure to prevent pathogens from entering into the plants (Oide et al., 2013; Eisenach et al., 2017). Nitrate reductase-catalyzed NO production is crucial for ABA-induced stomatal closure because the expression of key positive regulators in guard cell ABA signaling cascade was down-regulated in *nia1nia2* plants (Zhao et al., 2016). In agreement with our finding, GHPs, including 3BN, were reported to stimulate stomatal closure in Arabidopsis, a process accompanied by ROS and NO production as well as cytosolic Ca^2+^ fluctuation in the guard cells (Hossain et al., 2013).

The SA concentration in *nia1nia2* plants was relatively lower than in Col-0 plants under mock condition (Figure 9A), which suggests that SA production is stimulated by endogenous NO levels. Meanwhile, the increased production of SA in *nia1nia2* plants following the 3BN treatments, hints that NO and other secondary messengers, such as ROS, might act alone or synergistically to activate the 3BN-mediated SA signaling. The coordinated interplay between NO and SA has been characterized in detail (Freschi, 2013). For instance, sequence analysis on the promoters of NO-responsive genes in Arabidopsis revealed association with SA-responsive cis-regulatory elements (Palmieri et al., 2008). Moreover, NO has been reported to positively impact SA production and positively regulate SA signaling via two ways, i.e., (i) S-nitrosylation of non-expressor of pathogenesis-related proteins-1 (NPR1) to facilitate its oligomerization and interaction with SA receptors in cytosol, and (ii) S-nitrosylation of TGACG-Binding Factor-1 (TGA1) to stabilize the binding of the transcription factor to its downstream promoters (Mur et al., 2013). At the later bacterial infection stage, S-nitrosylation of SA-binding protein 3 (SABP3) inhibits its binding to SA, hence functioning as a negative feedback loop to suppress the activated SA signaling (Wang et al., 2009). Generation of ROS has also been associated with the activation of different phytohormone signaling pathways, including SA, that eventually lead to stress responses (Mohanta et al., 2018).

The greater induction of JA production in the *nia1nia2* plants is probably due to its lower endogenous SA level as compared to Col-0 plants (Figure 9A). This could be explained by the antagonistic interactions between JA and SA signaling (Takahashi et al., 2004). The fact that the different concentrations of 3BN treatments increased JA production by a greater fold change than SA production in both the Arabidopsis genotypes, warrants us to have a closer look at the interplay between NO and JA production/signaling. Consistent with our microarray data (Supplementary Tables S1, S2), NO has been reported to positively impact JA production by inducing the expression of its biosynthetic genes, such as lipoxygenase 3 (*LOX3*) and 12-oxophytodienoate reductase 1, 2, and 3 (*OPR1*, *2* and *3*) (Mur et al., 2012). On the other hand, JA signaling can be suppressed by NO via a number of ways, i.e., (i) S-nitrosylation of NPR1 in the cytosol, (ii) the induction of WRKY70 by monomeric NPR1 in the nucleus, and (iii) the binding between TGA factors and octadecanoid-responsive Arabidopsis59 (ORA59) promoter (Mur et al., 2012; Freschi, 2013). This delicate, coordinated and reversible interactions between NO and JA/SA signaling enable Arabidopsis to mount effective defense responses to pathogens with different modes of infection.

### The Disease Tolerance of Arabidopsis Against Necrotrophic Pathogens Is Enhanced by 2.0 mM 3BN Treatment

Exogenous application of 2.0 mM 3BN enhanced the disease tolerance of Arabidopsis Col-0 toward two types of necrotrophic pathogens, *Pectobacterium carotovorum* ssp. *carotovorum* (agent of bacterial root rot of sweet potato) (Figure 10) and *Botrytis cinerea* (agent of gray mold) (Figure 11). The infection assays of necrotrophic pathogens were carried out because 2.0 mM 3BN treatment increased the production of JA more than SA in Arabidopsis Col-0 plants (Figure 9). It is worth noting that the direct effect of 3BN on the pathogens could not be totally excluded even though precaution step had been taken by placing 3BN-treated plants in ventilated fume hood for 30 min to get rid of the 3BN vapor.

Based on the findings of this study, the proposed model of 3BN-induced plant defense responses in Arabidopsis involves potential key signaling compounds, NO, ROS, JA, and SA. NO is required for lesion formation (Figures 2, 3 and Supplementary Figure S2) and readjustment of metabolic processes involving the co-activation of sucrose degradation, glycolysis, Krebs cycle and amino acid synthesis (Figures 6–8 and Supplementary Figure S3) in Col-0 plants treated with 2.5 mM 3BN. This gaseous molecule is also involved in mediating the 3BN-induced SA and JA production in Col-0 plants at lower concentration of 3BN treatment (2.0 mM) (Figure 9A). SA and JA are well known as an important signaling molecule responsible for local hypersensitive response and activation of systemic acquired resistance (Ponce de León and Montesano, 2013; Gao et al., 2015). The activation of SA and JA signaling pathway might also be involved in transcriptional reprogramming that alters the primary metabolite profiles in Arabidopsis as part of the defense mechanisms against pathogens (Figures 6–8). Apart from the induction of broad defense responses in Arabidopsis reported in this study, it is crucial to identify the membrane-bound PRR that might bind 3BN in order to propose 3BN as DAMP (Li et al., 2020). As a promising first step in this endeavor, our transcriptomic data showed that the expression of 16 genes encoding protein kinases was induced by 2.5 mM 3BN treatment in Col-0 plants (Supplementary Table S2). They include lectin-receptor kinases (At1g69270, At2g37710, At3g59700), cell wall-associated kinase (WAK1; At1g21250), FLG22-induced receptor-like kinase 1 (FRK1; At2g19190); BAK1-interacting receptor-like kinase 1 (BIR1; At5g48380) and chitin elicitor receptor kinase 1 (CERK1; At3g21630).

## Conclusion

This study has demonstrated that exogenous application of 3BN, that belongs to the nitrile group of GHPs, can initiate an innate immune response by eliciting a broad range of signal molecules and pathways in Arabidopsis. At the concentration of 2.5 mM, 3BN treatment started to trigger a NO-mediated lesion formation in Arabidopsis. The same 3BN treatment also resulted in an elevated production of soluble sugars, Krebs cycle associated carboxylic acids and amino acids in Arabidopsis whose roles are closely related to the different stages of a plant immune response. Increased production of defense-related phytohormones such as SA, JA and ABA was observed in plants exposed to two different concentrations of 3BN treatments. As 3BN treatments of 2.5 mM and ≥ 5.0 mM caused lesion and severe necrosis respectively, a lower concentration of 3BN (2.0 mM) was used to test its feasibility in activating defense response of Arabidopsis without causing lesion formation. Intriguingly, this 3BN treatment also triggered an elevated synthesis of SA and JA. In the infection assay, 2.0 mM 3BN treatment was capable of enhancing the disease tolerance of Arabidopsis against necrotrophic pathogens such as *Pectobacterium carotovorum* ssp. *carotovorum* and *Botrytis cinerea*. In conclusion, this study proposes the potential of 3BN to function as DAMP in Brassicaceae.

## Data Availability Statement

The datasets generated for this study can be found in the GEO (accession GSE139089).

## Author Contributions

H-MT, AB, and RK conceived and designed the research project. H-MT, Y-CC, P-MY, AV, and RK conducted the experiments. H-MT, BC, C-PC, FY, AB, and RK contributed to data interpretation and manuscript preparation. JR contributed to metabolite analysis. PW contributed to microarray analysis. All authors have read and approved the final version of the manuscript.

## Conflict of Interest

The authors declare that the research was conducted in the absence of any commercial or financial relationships that could be construed as a potential conflict of interest.

## Footnotes

1 http://www.pantherdb.org/
