# Supplementary material for: The Role of a Glucosinolate-Derived Nitrile in Plant Immune Responses
Source: Front Plant Sci. 2020 Mar 10;11:257. doi: 10.3389/fpls.2020.00257 (PMC7076197; doi:10.3389/fpls.2020.00257)
Supplement: Supplementary file 1 [file Presentation_1.pdf]

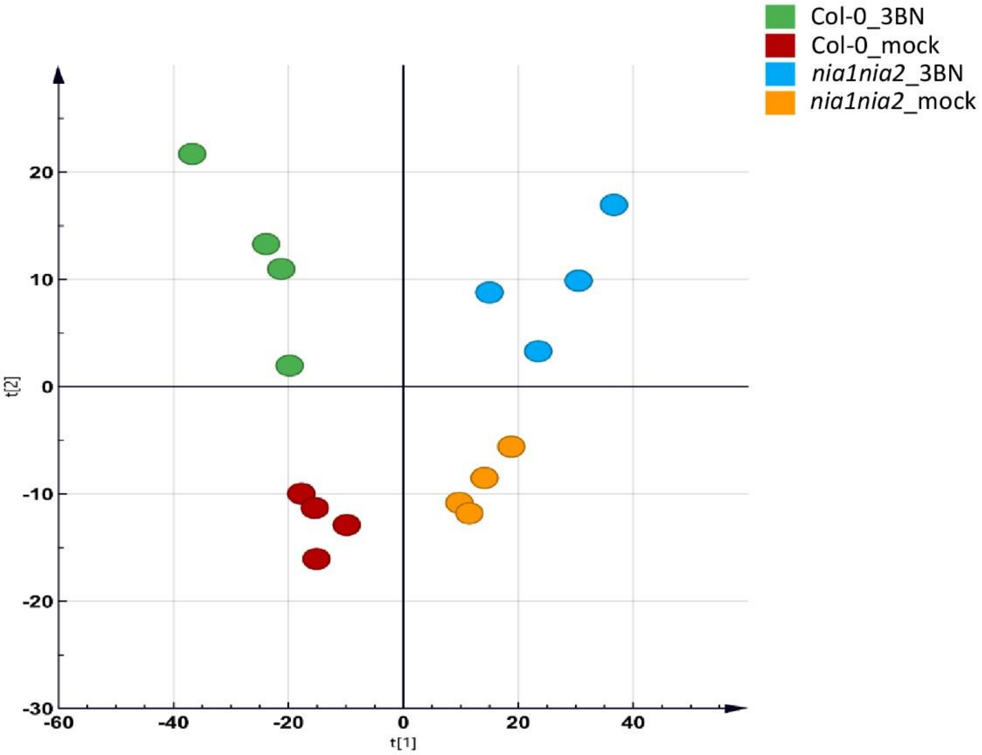

Supplementary Figure S1 | Principal component analysis (PCA) plot of the metabolite profiling samples. The first two principle components are plotted and colored according to Arabidopsis genotypes and experimental conditions.

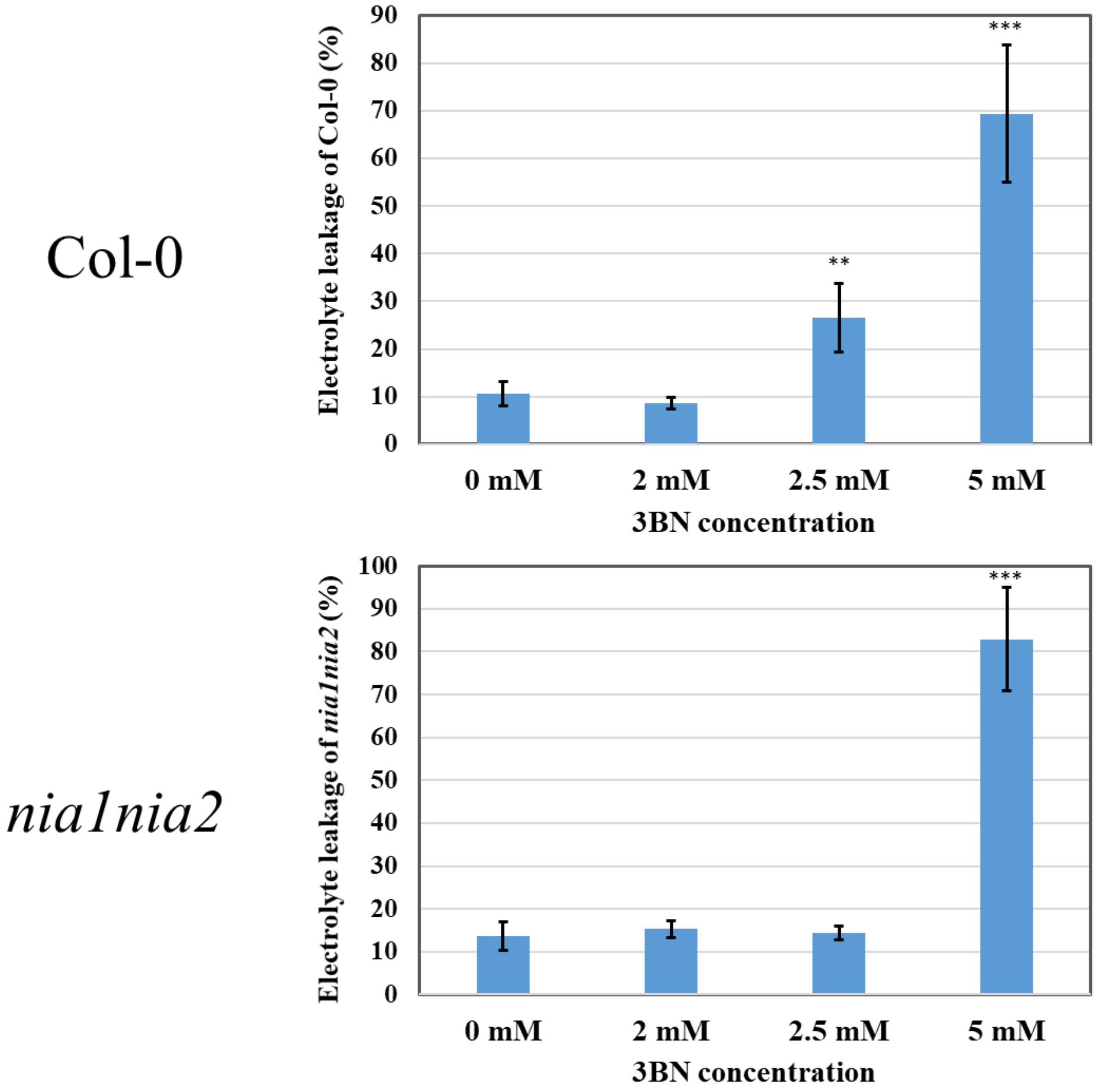

Supplementary Figure S2 | Electrolyte leakage analysis of Arabidopsis Col-0 wild-type and *nia1nia2* mutant when subjected to different concentrations of 3BN treatments (second trial; including 2.0 mM 3BN treatment). This analysis was carried out two-days post 3BN treatment. 2.5 mM 3BN treatment caused significant electrolyte leakage in wild-type but not in *nia1nia2* mutant. Values for electrolyte leakage are the average (error bars indicate SD) of six plants. Stars indicate a statistically significant difference (One-way ANOVA, **: $P < 0.01$, ***: $P < 0.001$) to the control treatment.

Col-0
(3BN/Mock)

nia1nia2
(3BN/Mock)

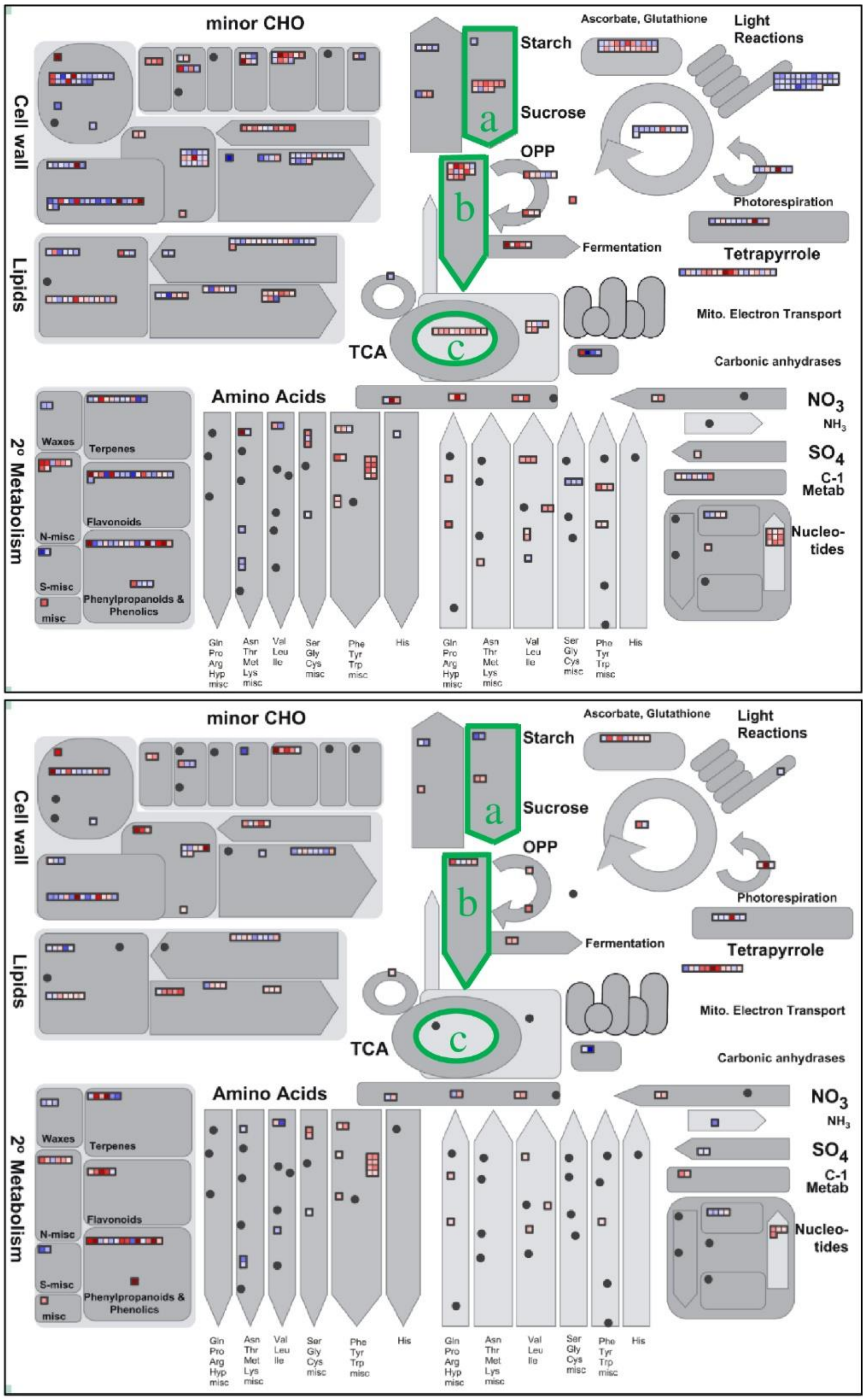

Supplementary Figure S3 | An overview of the metabolic processes differentially regulated between the Arabidopsis Col-0 and *nia1nia2* plants upon exposure to 24 hours of 2.5 mM 3BN treatment. The differentially regulated metabolic processes include (a) starch and sucrose metabolism, (b) glycolysis and (c) Krebs cycle. In all of the highlighted processes, more up-regulated genes were detected in the Col-0 plants compared with *nia1nia2* plants. DEGs are depicted with red and blue squares, where red color represents up-regulation and blue color represents down-regulation at $|\log_2 \text{fold change}| \geq 1$ and adjusted p-value <0.01. The corresponding gene lists are shown in Supplementary Table S4.
